# Leveraging Nanotechnology for Safer Herbicide Use: Insights from Maize Tolerance to Nanoencapsulated Atrazine

**DOI:** 10.1021/acsomega.5c04949

**Published:** 2025-08-20

**Authors:** Bruno Teixeira de Sousa, Bruno Henrique Bortotto da Silva, Euler Augusto Inêz, Anderson Do Espírito Santo Pereira, Jhones Luis Oliveira, Leonardo Fernandes Fraceto, Giliardi Dalazen, Halley Caixeta Oliveira

**Affiliations:** † Department of Animal and Plant Biology, 37894 State University of Londrina (UEL), Londrina, Paraná 86057-970, Brazil; ‡ Department of Agronomy, 37894 State University of Londrina (UEL), Londrina, Paraná 86057-970, Brazil; § B.nano Technological Solutions, Sorocaba, São Paulo 18078-005, Brazil; ∥ Institute of Science and Technology, São Paulo State University (UNESP), Sorocaba, São Paulo 18087-180, Brazil

## Abstract

Previous work has shown that nanoencapsulation of atrazine enhances the herbicidal action of this active ingredient. This increased activity is expected to control weeds and not compromise the tolerance of maize plants to the herbicide. This study aimed to evaluate the tolerance of maize plants to atrazine in postemergence application with different nanoformulations. Parameters of photosystem II (PSII) activity and growth of the maize plants in a greenhouse were evaluated after application of the formulations: conventional atrazine (ATZ), atrazine encapsulated in nanocapsules (NCs) of poly­(ε-caprolactone) (PCL + ATZ), PCL coated with chitosan (PCL/CS + ATZ), and zein (ZN + ATZ), at doses of 1000 and 2000 g active ingredient (a.i.) ha^–1^, in addition to the three NCs formulations without a.i. Oxidative stress markers and the activity of antioxidant and detoxification enzymes were quantified only in plants applied with atrazine, PCL + ATZ, PCL/CS + ATZ, and ZN + ATZ at a dose of 2000 g a.i. ha^–1^. All NC nanoformulations without a.i. were nontoxic to the physiology and growth of maize plants. Plants treated with ATZ developed up to 39% higher glutathione S-transferase activity and lower inhibition of PSII (on average 12%) compared to the nanoencapsulated herbicide. In contrast, PCL + ATZ, PCL/CS + ATZ, and ZN + ATZ induced higher antioxidant enzymatic activity in comparison to ATZ, like increases in ascorbate peroxidase (26%), catalase (95%), peroxidase (120%), and superoxide dismutase (41%). The effect on photosynthetic activity was transient for all formulations tested, with total recovery observed 14 days after application. Furthermore, ATZ, PCL + ATZ, PCL/CS + ATZ, and ZN + ATZ did not compromise maize plant growth. These results indicate that, under the evaluated conditions, maize plants remained tolerant to the active ingredient applied in a nanoencapsulated formulation.

## Graphical abstract

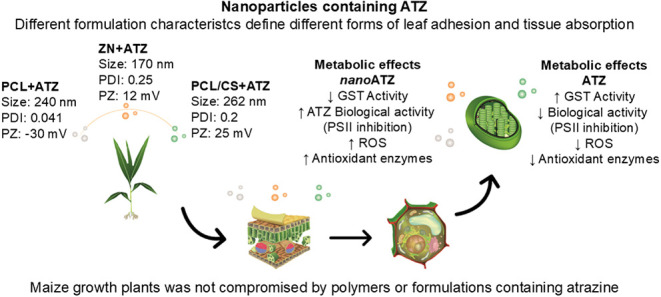

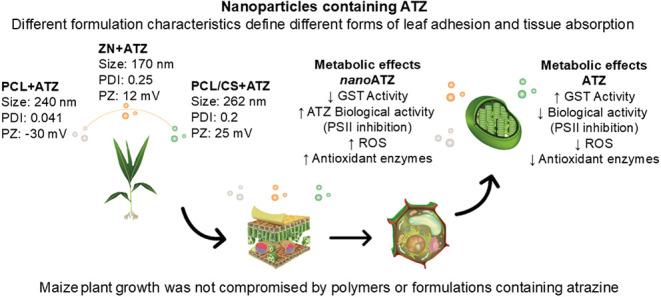

## Introduction

Nanotechnology can be applied to sustainable agriculture to produce food and raw materials with reduced environmental impacts.^1,2^ Nanotechnology tools or products can be used in different phases of plant cultivation and for various purposes, such as seed treatment, fertilization, control of weeds, insect pests, and pathogens (pesticides), postharvest and storage, biomonitoring, and bioremediation.^1–5^ The forms of nanomaterial application include nanocapsules (NCs), which are polymeric structures with an aqueous or oily core,^6^ that can act as nanocarriers by binding or encapsulating different active ingredients (a.i.).^7^ Three nanosystems for carrying the herbicide atrazine have been successfully developed and studied for over a decade. The first formulation obtained was composed of poly­(ε-caprolactone) (PCL) NCs,^8^ leading to increased postemergent herbicide efficiency and better control of different weed species under greenhouse and field conditions.^9–12^ Zein, a protein extracted from maize plants (*Zea mays* L.), was the second matrix used for atrazine nanoencapsulation, resulting in gains in efficiency in pre-emergent weed control.^13^ Recently, the formulation of PCL NCs containing atrazine was modified, adopting the chitosan coating strategy, further enhancing the postemergence herbicidal activity.^14^

The different polymers used in nanoencapsulation yield carrier systems with unique characteristics, including size, shape, zeta potential, composition, and dispersion of NCs, which directly influence their adhesion, absorption, and translocation, as well as the mechanisms of cellular internalization by plants.^15–17^ In the case of the atrazine nanocarriers, the zeta potential is negative in PCL NCs, and positive in those composed of zein and chitosan-coated PCL. Regarding the size, the NCs range from an average of 170 nm for NCs composed of zein, 240 nm for NCs based on PCL, and 260 nm in chitosan-coated PCL NCs.^8,13,14^ Furthermore, due to the different polymer compositions, these systems exhibit distinct release profiles of the active ingredient (a.i.).^8,13^

Atrazine is an herbicidal molecule which belongs to the triazines chemical group, that inhibits photosystem II (PSII) in plants, generating oxidative stress.^18,19^ The herbicide atrazine is one of the techniques employed in integrated weed management in maize production,^20^ one of the three most widely produced cereals globally.^21^ The known natural tolerance of maize plants to the a.i. atrazine is due to the detoxification metabolism of the molecule through the action of glutathione-*S*-transferase (GST) enzymes and nonenzymatic hydroxylation.^22,23^ Thus, weed control can be carried out without causing relevant damage to the maize plants.^24,25^ Atrazine use must be improved to reduce the adverse effects of environmental pollution caused by this herbicide molecule,^26,27^ which has been banned in several European countries and Uruguay.^28,29^ The nanoencapsulation of this a.i. using polymeric matrices can be a strategy for mitigating the negative environmental effects of the herbicide and for achieving better weed control compared to commercial products,^8,11,30^

Several challenges arise during the synthesis and application of an agrochemical carrier nanoformulation, ensuring that it is both safe and efficient.^31^ Specifically for nanoencapsulated atrazine formulations, enhancing the herbicidal action in weed control should not compromise the tolerance of maize plants to the herbicide. It has been demonstrated that postemergence application of up to 2000 g a.i. ha^–1^ of atrazine contained in PCL NCs led to low phytotoxicity transient inhibition of PSII, and did not compromise the development of maize plants in greenhouse ambient.^32^ When sown in pots 22 days after pre-emergent application of up to 2000 g a.i. ha^–1^ of atrazine contained in zein NCs, maize plants did not show inhibition of germination or PSII activity, nor changes in the evaluated growth parameters.^13^ However, a systematic comparison of how different polymeric matrices affect the tolerance of maize plants to atrazine has not been conducted yet.

In this sense, the objective of the current study was to evaluate the impacts of three atrazine carrier nanosystems on the tolerance of maize plants to the a.i., comparing them to the effects of applying the commercial formulation of atrazine. In addition, we evaluated the potential phytotoxicity of the polymers to maize plants by testing their variants without introducing a.i. The investigation was divided into two stages, the first involving evaluations of PSII inhibition and assessment of maize plant growth, and the second based on the biochemical responses of the plants to the application of the formulations.

## Results

### Inhibition of PSII and Plant Growth

The three nanoformulations containing atrazine, at both applied doses (D1: 1000 g a.i. ha^–1^; D2: 2000 g a.i. ha^–1^), reduced *F*_v_/*F*_m_ values in maize plants at 24 HAA (Figure 1a). In D1, the inhibitions of PSII activity to CTL were 10%, 16%, and 14% for plants that received the application of PCL + ATZ, PCL/CS + ATZ, and ZN + ATZ, respectively. In D2, the inhibitions were 18%, 19%, and 21% in plants which received PCL + ATZ, PCL/CS + ATZ, and ZN + ATZ, respectively. The *F*_v_/*F*_m_ of the plants that received the ATZ formulation differed from CTL only in D2, being 7% lower. For the PCL + ATZ and ZN + ATZ formulations, the PSII inhibitions in D2 were greater than in D1. The application of the formulations containing only the polymers, PCL, PCL/CS, and ZN, did not significantly reduce the *F*_v_/*F*_m_ of the maize plants.

**Figure 1:**
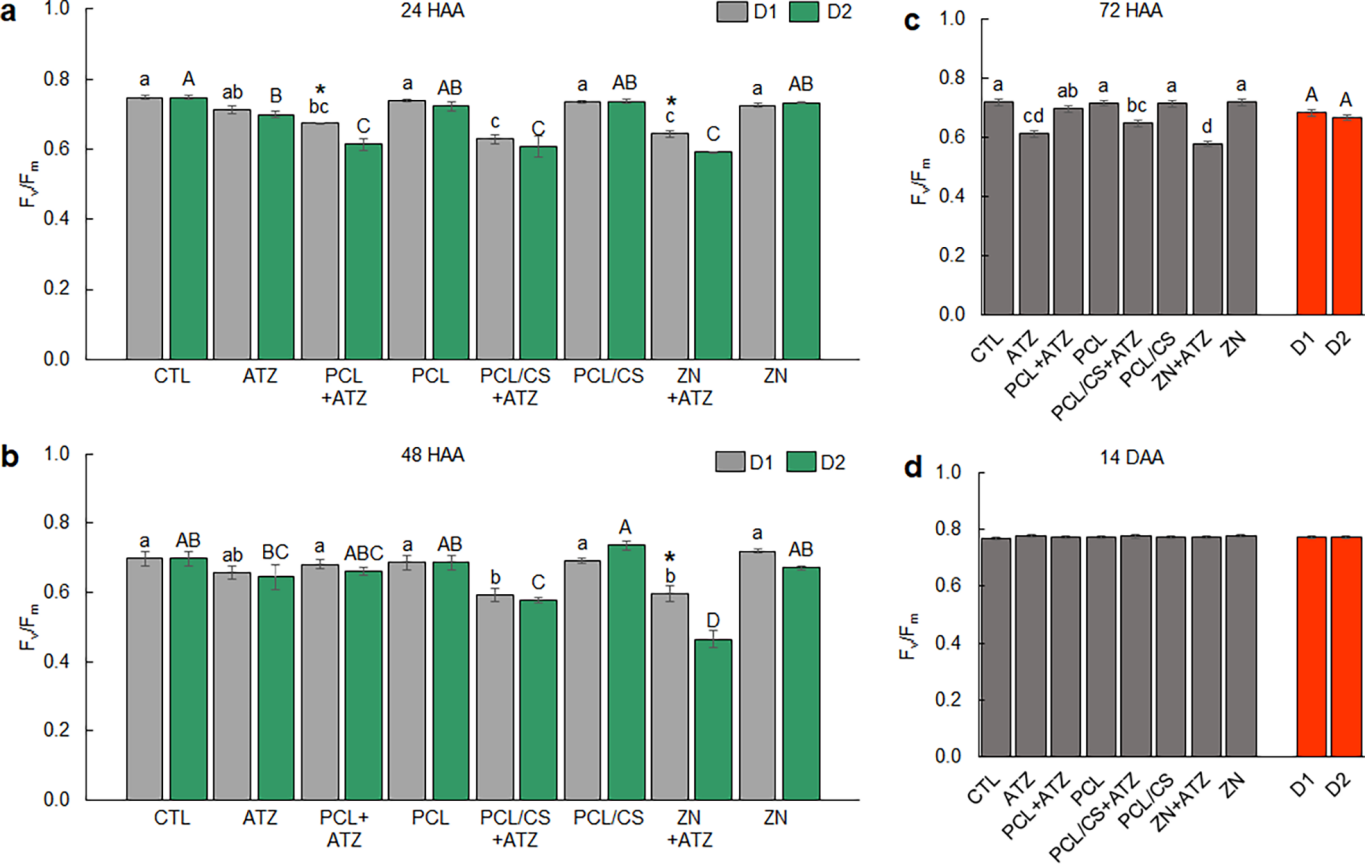
Maximum quantum yield of photosystem II (*F*_v_/*F*_m_) in maize in the periods of (a) 24, (b) 48, and (c) 72 h after application (HAA), and (d) 14 days after application (DAA) of ATZ, PCL + ATZ, PCL/CS + ATZ, or ZN + ATZ, at doses of 1000 g a.i. ha^–1^ (D1) and 2000 g a.i. ha^–1^ (D2), in addition to the nanocapsules without the a.i. in the same dilutions (PCL, PCL/CS, ZN) and control with only water (CTL). In (a) and (b), different lowercase letters indicate differences between formulations within D1; different uppercase letters indicate differences between formulations within D2; and asterisks indicate differences between doses of the same formulation by the Tukey test *p* ≤ 0.05. In (c), different lowercase letters indicate differences between treatments and different uppercase letters indicate differences between doses by the Tukey test *p* ≤ 0.05. In (d), that no significant effect was found by the F test (*p* > 0.05). Results are expressed as mean (*n* = 5) ± standard error.

At 48 HAA, only the PCL/CS + ATZ and ZN + ATZ formulations inhibited the PSII of maize plants, so the *F*_v_/*F*_m_ value differed from that of the CTL (Figure 1b). The inhibitions caused by PCL/CS + ATZ and ZN + ATZ were 15% and 15% in D1 and 17% and 33% in D2. There was a difference between doses for the *F*_v_/*F*_m_ value measured in maize plants only in ZN + ATZ, with the inhibition being greater at D2. At 72 HAA the dose–effect was lost, with similar *F*_v_/*F*_m_ values for both (Figure 1c). Regarding the formulations, only ATZ, PCL/CS + ATZ, and ZN + ATZ inhibited PSII, reducing the *F*_v_/*F*_m_ values to CTL, being 15%, 10%, and 20% lower, respectively. At 14 DAA, there was complete recovery of the maximum quantum yield of the PSII of the maize plants, with no further differences between treatments (Figure 1d).

The rETR of the maize plants was also compromised by the application of the PCL + ATZ, PCL/CS + ATZ, and ZN + ATZ formulations at 24 HAA, with reductions of 45%, 65%, and 59% to CTL, respectively (Figure 2a). There were no differences in rETR to the doses. At 48 HAA, the rETR of maize plants was reduced by the PCL/CS + ATZ formulations in D1 and ZN + ATZ in both doses (Figure 2b). The reductions with CTL were 54%, 43%, and 60%, respectively, for the mentioned treatments. At 72 HAA, all formulations at both doses reduced the rETR of maize plants about CTL (Figure 2c). In this evaluation, the application of the ATZ formulation resulted in rETR at a level similar to that of the nanoformulations. The formulations ATZ in D1 (37%), PCL/CS + ATZ in D2 (37%), and ZN + ATZ in both doses (49% and 64%, respectively) stood out as having the greatest inhibition of rETR. At 14 DAA, as in *F*_v_/*F*_m_ the recovery of the PSII activity of the maize plants is visible, however, only plants that received application of the PCL and ZN formulations presented rETR rates similar to CTL, with small reductions in the other formulations (Figure 2d).

**Figure 2:**
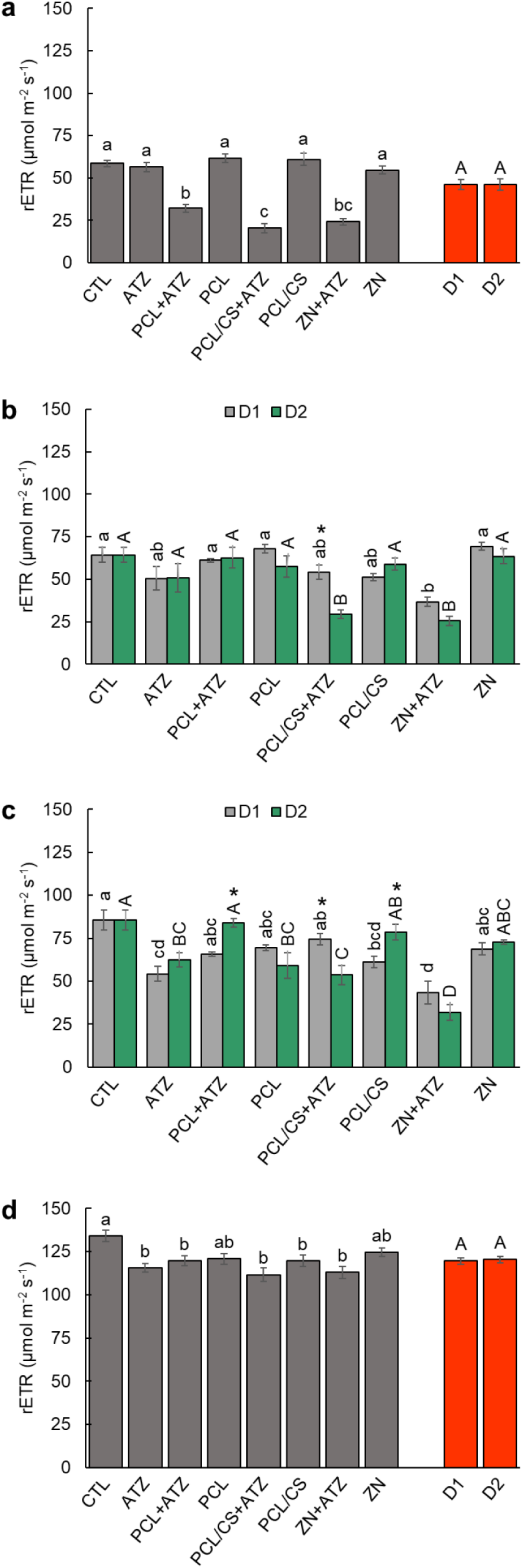
Relative electron transport rate (rETR) in maize during the periods of (a) 24, (b) 48, and (c) 72 h after application (HAA), and (d) 14 days after application (DAA) of ATZ, PCL + ATZ, PCL/CS + ATZ, or ZN + ATZ, at doses of 1000 g a.i. ha^–1^ (D1) and 2000 g a.i. ha^–1^ (D2), in addition to the nanocapsules without the a.i. in the same dilutions (PCL, PCL/CS, ZN) and control with only water (CTL). In (a) and (b), different lowercase letters indicate differences between formulations within D1; different uppercase letters indicate differences between formulations within D2; and asterisks indicate differences between doses of the same formulation by the Tukey test *p* ≤ 0.05. In (c) and (d), different lowercase letters indicate differences between treatments and different uppercase letters indicate differences between doses by the Tukey test *p* ≤ 0.05. Results are expressed as mean (*n* = 5) ± standard error.

For the stomatal conductance (*g*_s_) of maize plants, no formulation reduced the values of *g*_s_ evaluated at 48 HAA (Figure S1a). In the evaluation at 72 HAA, only the application of D1 of ATZ led to a reduction in *g*_s_ to CTL plants (29%), while the application of D2 of ZN promoted a 67% increase in *g*_s_ of maize plants with CTL (Figure S1b).

Plants that received application of ATZ formulations at dose D2 and ZN + ATZ at dose D1 showed fewer expanded leaves (Figure 3a). However, the application of PCL/CS + ATZ and PCL/CS formulations at dose D2 and ZN + ATZ at dose D1 promoted higher averages of expanded leaves to CTL. The ATZ and PCL/CS + ATZ formulations showed a lower number of expanded leaves at dose D2, while in ZN + ATZ this effect was found at dose D1. The stem diameter of maize plants (Figure 3b) was increased by between 5% and 7% in relation to the CTL after applying dose D1 of all formulations, except in ZN + ATZ. At dose D2, only PCL and ZN + ATZ promoted increases in stem diameter of 21% and 17% respectively. The ZN + ATZ formulation was the only one to show a difference in stem diameter between doses, with dose D2 providing stems with an average diameter 20% smaller than dose D1. Maize plant height was not affected by any formulations at dose D2, while at dose D1 it was increased by PCL by 14% and PCL/CS by 3% compared to CTL (Figure S2a). Maize plant root growth, regardless of dose, was promoted by all formulations (between 4% and 16%), except for PCL/CS, which did not differ from CTL (Figure S2b).

**Figure 3:**
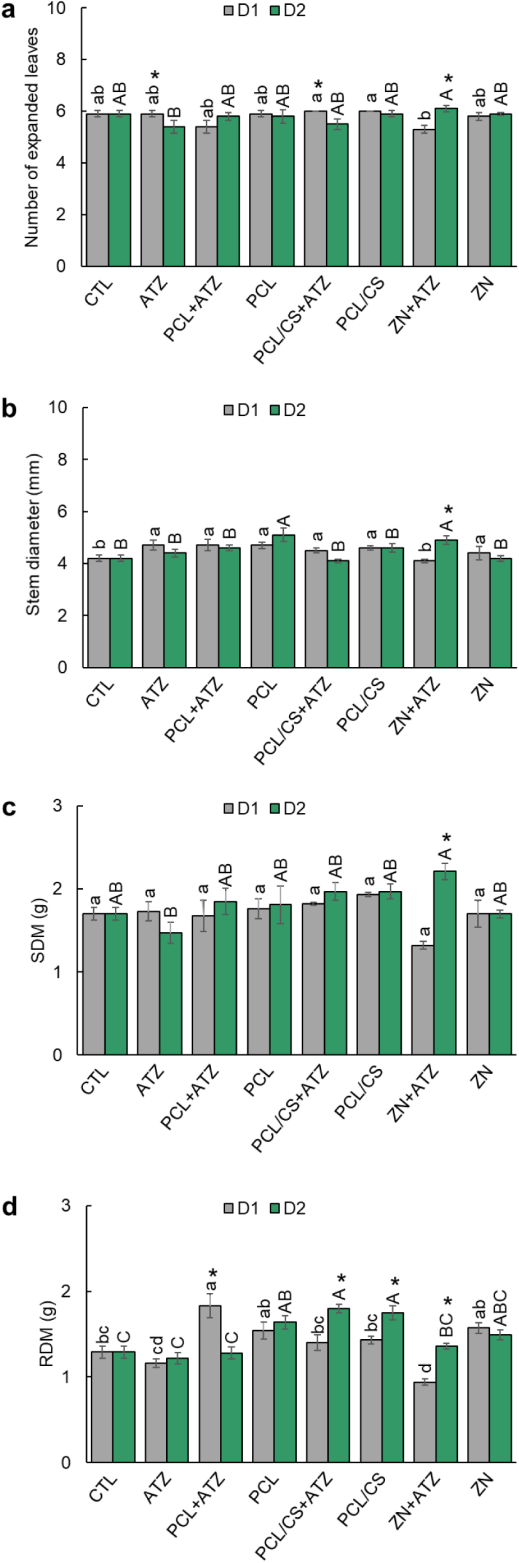
(a) Number of expanded leaves, (b) stem diameter, (c) shoot dry mass (SDM), and (d) root dry mass (RDM) of maize 30 days after application (DAA) of ATZ, PCL + ATZ, PCL/CS + ATZ, or ZN + ATZ, at doses of 1000 g a.i. ha^–1^ (D1) and 2000 g a.i. ha^–1^ (D2), in addition to the nanocapsules without the a.i. in the same dilutions (PCL, PCL/CS, ZN) and control with water only (CTL). Different lowercase letters indicate differences between formulations within D1; different uppercase letters indicate differences between formulations within D2; and asterisks indicate differences between doses of the same formulation by the Tukey test *p* ≤ 0.05. Results are expressed as mean (*n* = 5) ± standard error.

The shoot dry mass of the maize plants (Figure 3c) was not altered by dose D1 of any formulations, while at dose D2 it was reduced by ATZ by 14% and increased by ZN + ATZ by 30% (in relation to CTL). Only the ZN + ATZ formulation differed in doses, with a lower dry mass of plants at dose D1, 67% lower in relation to dose D2. For root dry mass (Figure 3d), within the D1 dose, the masses were reduced by ATZ and ZN + ATZ by 10% and 27%, respectively, in relation to CTL, and increased by PCL + ATZ, PCL, and ZN by 41%, 19%, and 21%, respectively. Within the D2 dose, the formulations promoted mass gains of between 5% and 40%, except for the ATZ and PCL + ATZ formulations, which did not differ from the CTL. The PCL + ATZ formulation show demonstrated a 43% reduction in root mass at the D2 dose compared to the D1 dose. The PCL/CS + ATZ, PCL/CS, and ZN + ATZ formulations led to lower average dry mass of the roots at the D1 dose, being respectively 29%, 22% and 31% lower than in D2.

In this first investigation, it was observed that applying only the polymers used for the synthesis of NCs (PCL, and PCL combined with chitosan and zein) did not cause inhibition of PSII activity nor compromise the growth of maize plants. Based on these observations, only the NC formulations associated with the active ingredient atrazine were used to investigate biochemical responses to their application.

### Oxidative Stress and Enzyme Activity

The amount of hydrogen peroxide (H_2_O_2_) in the leaves of maize plants did not differ between treatments at 24 HAA (Figure 4a). At 48 HAA, only ATZ did not differ in the amount of H_2_O_2_ to CTL, while PCL + ATZ, PCL/CS + ATZ, and ZN + ATZ promoted increases of 32%, 31% and 22%, respectively. At 72 HAA, only the PCL + ATZ formulation did not differ from CTL, thus, ATZ, PCL/CS + ATZ, and ZN + ATZ represented increases of 23%, 33% and 18% in the accumulation of H_2_O_2_.

**Figure 4:**
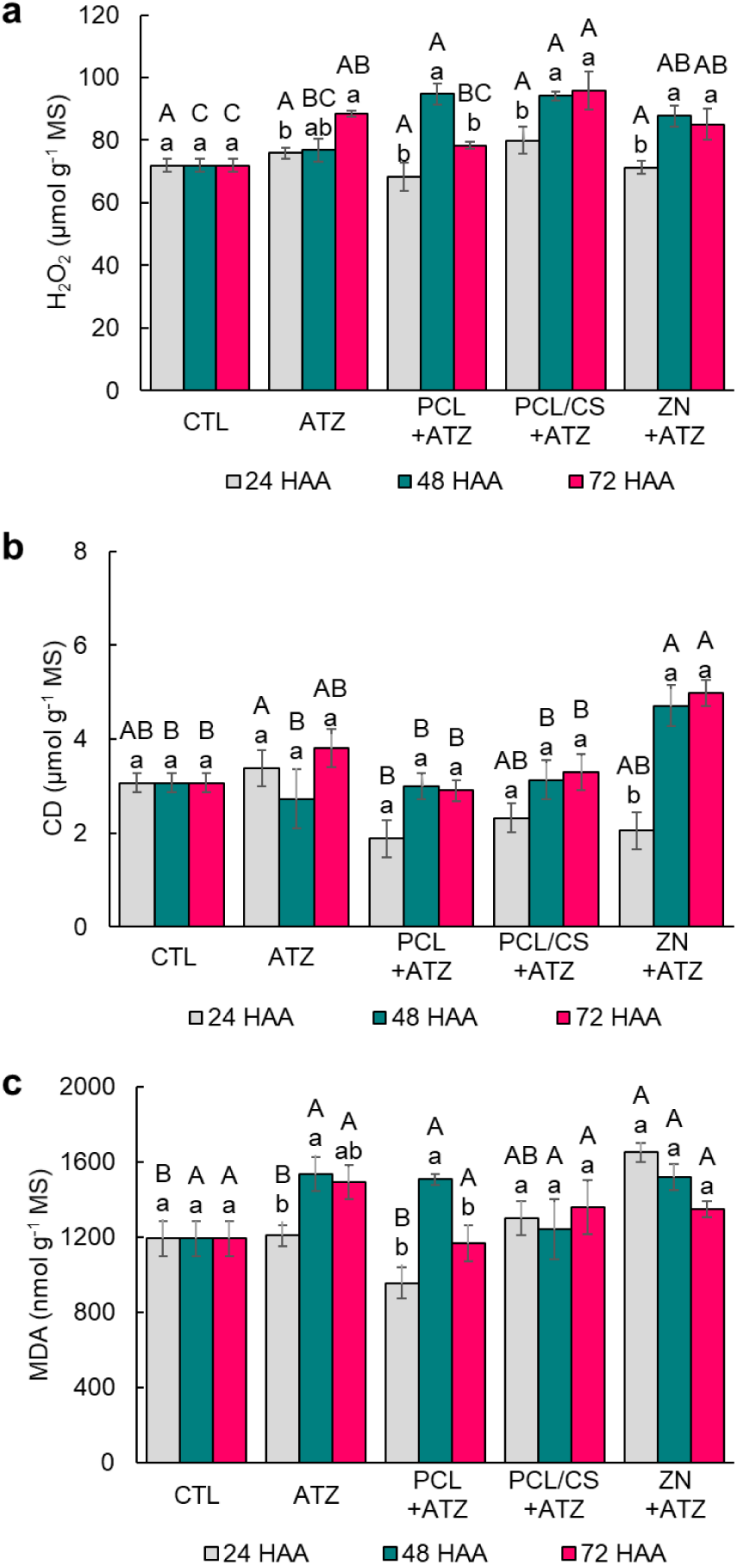
Amount of (a) hydrogen peroxide—H_2_O_2_, (b) conjugated dienes—CD, and (c) malondialdehyde—MDA in maize leaves at 24, 48, and 72 h after application (HAA) of ATZ, PCL + ATZ, PCL/CS + ATZ, or ZN + ATZ at a dose of 2000 g a.i. ha^–1^. Different lowercase letters indicate differences between collection times for the same treatment, and different uppercase letters indicate differences between treatments within the same collection time by the Tukey test *p* ≤ 0.05. Results are expressed as mean (*n* = 4) ± standard error.

The amount of conjugated dienes (CD) in the leaves of plants treated with the four formulations was like the CTL at 24 HAA (Figure 4b), and the only difference found was the lower amount of CD in plants treated with PCL + ATZ compared to ATZ (44%). At 48 HAA, the amounts of CD in plants that received ZN + ATZ were 54%, 73%, 57%, and 50% higher than those in CTL and in plants that received ATZ, PCL + ATZ, and PCL/CS + ATZ, respectively. At 72 HAA, the amounts of CD in plants treated with ZN + ATZ were higher than those in CTL, PCL + ATZ, and PCL/CS + ATZ by 62%, 71%, and 51%, respectively, and were similar to those in ATZ. A high increase in the amount of CD (about 130%) was observed in plants that received ZN + ATZ application between 24 to 48 HAA.

For the amount of malondialdehyde (MDA) in the leaves of maize plants, at 24 HAA the ZN + ATZ formulation promoted an increase of 38% in relation to CTL and 37% and 73% above those applied with ATZ and PCL + ATZ (Figure 4c). In the evaluations at 48 and 72 HAA, there was no difference between the treatments for the amount of MDA found in the leaves of the maize plants. The applications of ATZ and PCL + ATZ formulations resulted in a peak amount of MDA at 48 HAA, while in the plants that received PCL + ATZ and ZN + ATZ, there was no difference.

The activity of the enzyme glutathione S-transferase (GST) in the leaves of maize plants with 24 HAA of the four formulations was like CTL. Still, in the leaves of plants with ZN + ATZ the GST activity was 30%, 32% and 23% lower than in ATZ, PCL + ATZ, and PCL/CS + ATZ, respectively (Figure 5a). At 48 HAA, only the GST activity in leaves treated with ATZ differed from CTL, with increases of 39%, 23%, and 36% recorded over PCL/CS + ATZ, ZN + ATZ, and CTL, respectively. At 72 HAA, GST activity in the leaves of plants applied with ZN + ATZ was the lowest among all treatments, 38%, 28%, 41% and 32% lower than CTL, ATZ, PCL + ATZ and PCL/CS + ATZ. For the application of ATZ and ZN + ATZ, peaks of GST activity were recorded at 48 HAA, while in the other formulations, there was no difference between the evaluations.

**Figure 5:**
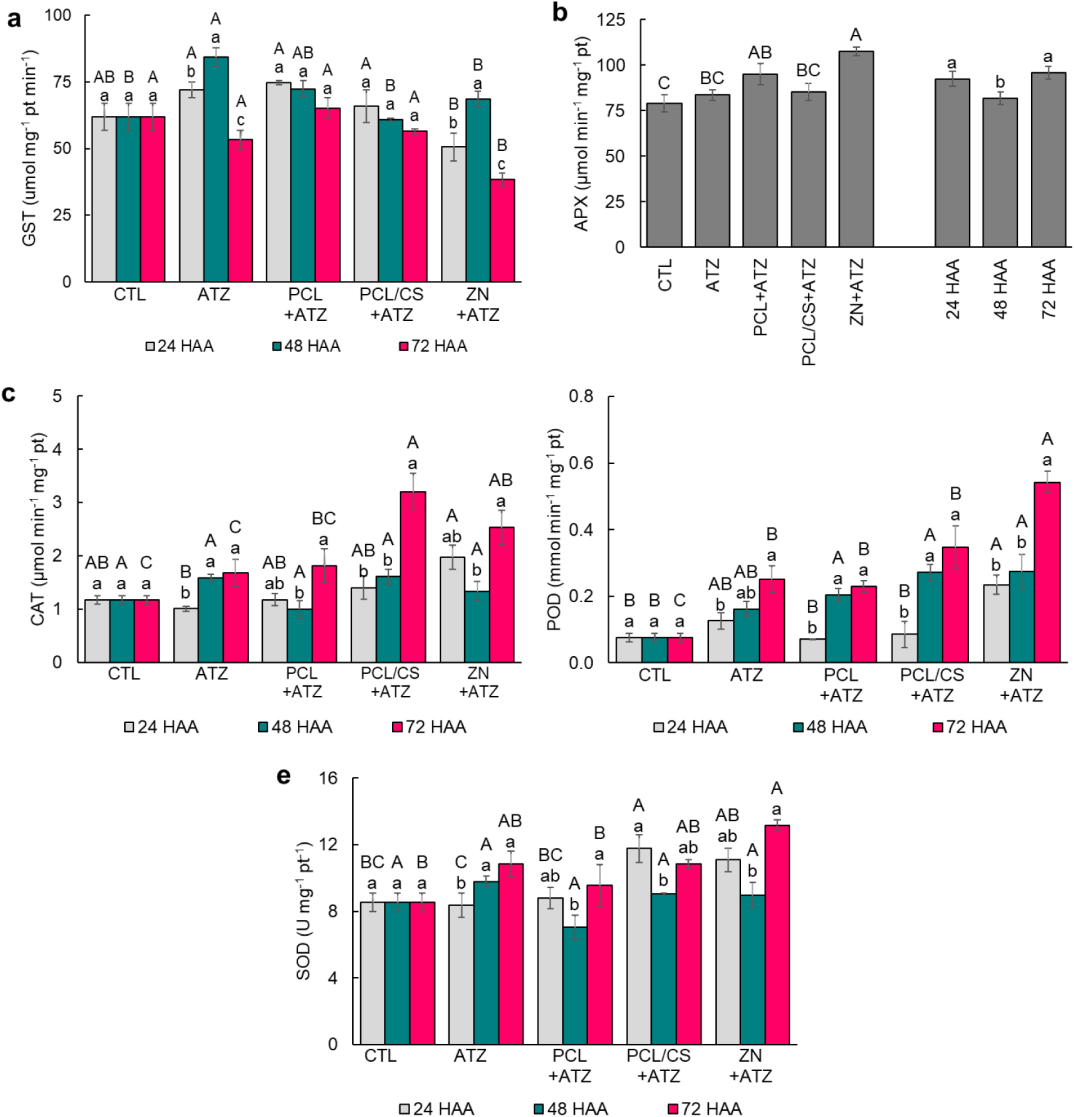
Activity of (a) glutathione *S*-transferase (GST), (b) ascorbate peroxidase (APX), (c) catalase (CAT), (d) peroxidases (POD), and (e) superoxide dismutase (SOD) in maize leaves at 24, 48, and 72 h after application (HAA) of ATZ, PCL + ATZ, PCL/CS + ATZ, or ZN + ATZ at a dose of 2000 g a.i. ha^–1^. In (a), (c), (d), and (e), different lowercase letters indicate differences between collection times for the same treatment, and different uppercase letters indicate differences between treatments within the same collection time by the Tukey test *p* ≤ 0.05. In (b), different lowercase letters indicate differences between collection times, and different uppercase letters indicate differences between treatments by the Tukey test *p* ≤ 0.05. Results are expressed as mean (*n* = 4) ± standard error.

For the enzyme Ascorbate Peroxidase (APX), regardless of the evaluation time, the activity in leaves applied with ZN + ATZ was 36% higher than that in CTL leaves, and 29% and 26% higher than those used with ATZ and PCL/CS + ATZ, respectively (Figure 5b). The APX activity in the leaves of plants applied with ATZ and PCL/CS + ATZ did not differ from that of CTL plants. Regarding the evaluation times, regardless of the treatment, the lowest APX activity in maize leaves occurred at 48 HAA. The activity of the enzyme Catalase (CAT) at 24 HAA in the leaves of plants of the four formulations was similar to CTL, and the only difference occurred between the plants that received application of ZN + ATZ, with activity 95% higher than that of those which received ATZ (Figure 5c). At 48 HAA, there was no difference between treatments for CAT activity. However, at 72 HAA, in leaves of plants applied with PCL/CS + ATZ and ZN + ATZ, CAT activity was higher than in the other treatments, while in ATZ and PCL + ATZ it did not differ from CTL. Over time, gradual increases in CAT activity were found after the application of all formulations.

The activity of Peroxidase (POD) enzymes in the leaves of maize plants at 24 HAA did not differ between the CTL, ATZ, PCL + ATZ, and PCL/CS + ATZ treatments, with POD activity in leaves applied with ZN + ATZ being 2 times higher than that of CTL, and 2.3 and 1.8 times higher than PCL + ATZ and PCL/CS + ATZ, respectively (Figure 5d). At 48 HAA, the POD activity in the leaves of plants applied with PCL + ATZ, PCL/CS + ATZ, and ZN + ATZ were 1.7, 2.6, and 2.6 times higher than CTL. At 72 HAA, the highest POD activity in maize leaves occurred in those applied with the ZN + ATZ formulation, and was 6 times higher than CTL and 1.2, 1.4 and 0,5 times higher than ATZ, PCL + ATZ and PCL/CS + ATZ. In the leaves that received application of ATZ, PCL + ATZ, and PCL/CS + ATZ also were higher than CTL, 2.3, 2, and 3.6 times, respectively. The POD activity increased over time after the application of all formulations.

For Superoxide Dismutase (SOD) activity, at 24 HAA, only the plants that received PCL/CS + ATZ application differed from CTL (Figure 5e). Also, SOD activity in leaves applied with PCL/CS + ATZ was 38% higher than CTL and 41% and 34% higher than in leaves applied with ATZ and PCL + ATZ. At 48 HAA, there was no difference in SOD activity between treatments. In the final evaluation, at 72 HAA, only the SOD activity in leaves applied with ZN + ATZ differed to the CTL plants, with a 54% increase in activity. Over time, after application, in the leaves of maize plants that received ATZ, there was a growing increase in SOD activity, whereas for the other formulations, there was a decline in activity at the 48-h assessment.

## Discussion

The three NCs formulations containing atrazine applied to maize plants provided the highest percentages of inhibition of PSII activity (*F*_v_/*F*_m_ and rETR) in the initial evaluation periods; however, the only adverse effect found on the morphological variables was a slight reduction in root dry mass provided by the ZN + ATZ nanoformulation (at a dose of 1000 g a.i. ha^–1^). Rapid recovery from PSII inhibition resulting from atrazine application is a characteristic found in maize plants. In the study by Jachetta and Radosevich,^44^ the 25% reduction in photosynthesis of maize plants resulting from atrazine application was recovered in just 21.4 h (measurements made by infrared gas analyzer). As reported in *Digitaria insularis* plants (an atrazine-tolerant species like maize), PCL + ATZ NCs potentiated the action of atrazine, increasing *F*_v_/*F*_m_ inhibition by approximately 50% compared to the conventional formulation.^11^ The rapid penetration and, consequently, the rapid delivery of atrazine at the site of action, as well as its greater entry through stomata and hydathodes, enhance the action of the herbicide,^12–14,45^ The slow and gradual release of the active ingredient by zein NCs,^46^ probably led to constant PSII inhibition percentages for a longer period than the other nanoformulations.

The application of polymeric NCs nanoformulations without active ingredient did not compromise the PSII activity of maize plants, and in some morphological parameters, stimulation of plant growth was observed. Similar results were obtained by applying unloaded PCL NCs in maize, where no inhibition of *F*_v_/*F*_m_, photosynthesis or plant dry mass was found.^32^ As a differential, the chitosan present in the PCL/CS + ATZ formulation can help mitigate the adverse effects of abiotic stresses.^47^ Zein NCs without active ingredients were nontoxic to bean plants (*Phaseolus vulgaris*) without reductions in the aerial part or root mass.^48^ Zein NCs have been tested on a large scale in combination with other drugs, mainly due to their low or no cytotoxicity.^49,50^

The enhancement of PSII inhibition by nanoatrazine also resulted in a greater amount of H_2_O_2_ (a reactive oxygen species), Plants that received application of PCL + ATZ, PCL/CS + ATZ, and ZN + ATZ presented at 48 HAA levels of H_2_O_2_ found in plants treated with ATZ only at 72 HAA. Although not quantified in the present work, nanoatrazine formulations generate a greater amount of O_2_^**•**–^ in plants, as already reported in lettuce plants (*Lactuca sativa*) in prolonged exposure to PCL + ATZ via soil.^51^ The greater amount of conjugated dienes (hydroperoxides resulting primarily from the attack of ROS membranes in the lipid peroxidation process^52^ found in plants treated with ZN + ATZ is possibly linked to the prolonged period of PSII inhibition in plants treated with this formulation.

The amount of MDA (compound formed as a secondary product of lipid peroxidation^52^) found in the leaves of maize plants after the application of ATZ, PCL + ATZ, and ZN + ATZ was higher than the CTL, also indicating the occurrence of lipid peroxidation and consequent physiological damage to the plants. Results similar to those reported by Oliveira et al.^32^ were obtained after the application of 2000 g a.i. of ATZ and PCL + ATZ in maize plants.

It has been suggested that lipid peroxidation is a continuous process, with high turnover and autocatalysis.^53^ The generation of oxidative stress was less significant in plants that received ATZ, in which the greatest detoxifying activity via GST was found (with peak activity at 48 HAA) in relation to the application of nanoformulations. Some plant species have a natural capacity to detoxify abiotic organic compounds (xenobiotics), such as herbicides, before their metabolic activation.^54,55^ The severity of the toxicity caused by atrazine in maize plants may be directly related to the efficiency and activity of the ^1^O_2_ inactivation and/or elimination system.^56^

In general, all formulations containing atrazine applied to plants promoted changes in the activity of enzymes with antioxidant action to CTL (without application). It has already been reported in the literature that in maize plants the application of atrazine tends to lead to an increase in the activity of antioxidant enzymes, such as APX, CAT, POD, and SOD.^57^ APX activity was higher in plants that received ZN + ATZ or PCL/CS + ATZ, regardless of the evaluation time after application. The increase in POD activity is directly related to the severity of stress faced by the plants.^19^

For CAT activity, the four formulations promoted gradual increases in activity between the three periods evaluated; however, the highest activities were observed in plants treated with PCL/CS + ATZ or ZN + ATZ at 72 HAA. The inhibition of CAT enzyme activity in plants of the species *Pennisetum americanum* was reported to result from increased atrazine concentrations.^58^ However, increased CAT activity is also associated with severe oxidative stress conditions, characterized by elevated concentrations of H_2_O_2_.^59^ Based on the facts that the four formulations were applied at the same dose (2000 g a.i. ha^–1^), as there was an increase in the amount of H_2_O_2_ in the plants of the four treatments, it can be suggested that the increase in CAT activity is related both to the reduction in the metabolization of atrazine molecules over time, and to the rise in the amount of.H_2_O_2_ that occurred.

Regarding SOD activity, in plants that received ATZ there was an increase in activity between the three periods evaluated, while in plants that received the application of the PCL + ATZ, PCL/CS + ATZ, and ZN + ATZ formulations there was a decline in activity at 48 HAA. In the final evaluation, the highest SOD activity was observed in plants that received ZN + ATZ. In plants of the species *Setaria itálica*, *Pennisetum americanum*, and *Arabidopsis thaliana*, increasing doses of atrazine inhibited the expression of SOD enzyme genes,^56,58,60^ however, in rice plants (*Oryza sativa*) SOD activity was increased with exposure to atrazine.^61^ The oscillations in SOD activity found in maize plants after application of different formulations are related to the increase in the concentration of atrazine molecules in the metabolism of maize plants at various flow rates.

In lettuce plants (*L. sativa*) grown in soil containing PCL + ATZ, the activity of APX, CAT, POD, and SOD was greater than in plants grown in soil containing ATZ at the same dose.^51^ As well as the greater effect on PSII inhibition, the application of atrazine nanoformulations also resulted in a higher level of oxidative stress in maize plants with significant activation of antioxidant defense mechanisms. In this sense, it can be observed that when the ATZ formulation was applied to maize plants, the activation of atrazine detoxification mechanisms occurred first as a defense, preventing the metabolic activation of the molecule and the consequent inhibition of PSII. With the application of ZN + ATZ and other nanoatrazine formulations, maize plants appeared not to recognize the atrazine molecule, allowing it to reach the site of action, undergo metabolic activation, and inhibit PSII, thereby triggering the antioxidant enzyme system as a defense against the oxidative stress caused. Thus, it is suggested that the alterations in the mode of action of atrazine after its application in plants are related to how the active ingredient is delivered to plants and its arrival at the site of action, resulting in a higher level of oxidative stress.

Minor adverse effects have been observed in maize morphology following the application of the three atrazine nanoformulations. Similarly, the absence of toxic effects resulting from atrazine encapsulation in chitosan-alginate NCs has been reported for maize plants.^62^ In the work of Munhoz-Garcia et al.,^63^ glyphosate-tolerant soybean (*G. max*) and cotton (*Gossypium hyrsitum*) plants did not show symptoms of toxicity after the application of the herbicide encapsulated in zein/poloxamer NCs. According to the authors, the absence of toxic effects in tolerant cultures following nanoherbicide treatment is a key factor in increasing weed control without compromising selectivity.

Nanoparticles generally interact with the surface of leaves, so that the electrical charges, size, and polymer matrix directly influence the absorption dynamics.^15,16,31,64,65^ The PCL + ATZ formulation comprises NCs with a negative electrical charge (zeta potential), around −30 mV. In contrast, the PCL/CS + ATZ and ZN + ATZ formulations comprise NCs with a positive zeta potential, approximately 25 mV and 12 mV, respectively. The positive zeta potential characteristic of the NCs of the PCL/CS + ATZ formulation is acquired after coating. In maize plants, it was observed that positively charged nanoparticles have a greater ease of reaching the chloroplasts than negatively charged nanoparticles and an easier entry through the stomata.^66^ The outer layer of the leaves is formed by epicuticular wax, which forms long-chain hydrocarbons with functional groups such as alcohols, aldehydes, and fatty acids, which gives the appearance of negative surface electrical charges.^67^ This characteristic favors the adhesion of positive potential NCs to the leaves,^15^ as in PCL/CS + ATZ and ZN + ATZ. Hydrophobic and hydrophilic interactions also interfere with adhesion and absorption.^16,31^ PCL + ATZ NCs have an amphiphilic character, with a higher contact angle and lower leaf wettability.^12^

The current study observed that the PCL, and PCL with chitosan and zein coating polymers used in the nanoencapsulation process did not compromise the physiology and growth of maize plants up to 30 days after application. Thus, using these polymers is not a limiting factor for the continued development of atrazine carrier nanosystems. When combined with atrazine, despite the greater initial inhibition of PSII caused, the maize plants presented a recovery process and their growth was not interrupted. In general, more significant biochemical signs of oxidative stress resulting from the action of atrazine were observed in plants that applied nanoatrazine formulations; however, enzymatic responses to combat abiotic stress were also triggered. Further investigations are required to assess the safety of field applications and the interactions of nanoformulations with soil microbiota.

## Methods

### Plant Material and Growing Conditions

Hybrid corn seeds GNZ 7740 VIP3 (Geneze Sementes), which have an early development cycle, were used. The seeds were sown directly in pots filled with Eutric Nitisol clayey latosol soil collected from the Experimental Farm of the State University of Londrina (UEL). The soil has a high clay content typical for northern Paraná, with chemical characteristics as described: pH (CaCl_2_), 4.83; organic matter, 28.2 g dm^–3^; P, 7.63 mg dm^–3^; K, 0.65 cmol_c_ dm^–3^; Na, 0.0 cmol_c_ dm^–3^; Ca, 3.96 cmol_c_ dm^–3^; Mg, 1.80 cmol_c_ dm^–3^; sum of bases, 6.41 cmol_c_ dm^–3^; cation exchange capacity at pH 7.0 (CEC), 11.0 cmol_c_ dm^–3^; and base saturation (BS), 58.2%. Base saturation was calculated using eq 1.

1 $$\mathrm{BS}=\left(\frac{K+\mathrm{Ca}+\mathrm{Mg}}{\mathrm{CEC}}\right)\times 100\;[\%]$$

Two plants were grown per pot in a greenhouse at the Center for Biological Sciences (CCB) of UEL. The formulations were applied to plants with four fully expanded leaf (stage V4) according to the Ritchie, Hanway, and Benson.^33^

### Formulations

Conventional Atrazine (ATZ): the formulation considered conventional (nonencapsulated) was sourced from the commercial product Primóleo (SC 400 g a.i. L^–1^, Syngenta) diluted to 1 g a.i. L^–1^.

Atrazine in poly­(epsilon-caprolactone) NCs (PCL + ATZ): synthesized according to Grillo et al.^8^ The formulation at a concentration of 1 g a.i. L^–1^ is composed of spherical NCs, with uniform distribution and no aggregates, with sizes between 240 ± 4 nm, a polydispersity index of 0.041 ± 0.05, zeta potential of −30 ± −2 mV, and nanoencapsulation efficiency of approximately 94% (Figure S3).

Atrazine in chitosan-coated poly­(epsilon-caprolactone) NCs (PCL/CS + ATZ): synthesized according to the protocol established by Sousa et al.^14^ The formulation at a concentration of 1 g a.i. L^–1^ is composed of spherical NCs, uniformly distributed, without aggregates, with sizes between 262 ± 3 nm, a polydispersity index closes to 0.2, zeta potential of 25 ± 2 mV, and nanoencapsulation efficiency of approximately 94% (Figure S3).

Atrazine in zein NCs (ZN + ATZ): synthesized according to the protocol of Carvalho et al.^13^ The nanoformulation at a concentration of 1 g a.i. L^–1^ is composed of spherical NCs, with sizes between 130 and 170 nm, a polydispersity index lower than 0.25, zeta potential around 12 mV, and encapsulation efficiency greater than 90% (Figure S3).

NCs without a.i.: the three herbicide nanoformulations were also synthesized without the addition of the a.i. (following the same synthesis protocols already described), these will be called PCL, PCL/CS, and ZN.

### Stage 1 – Physiological and Growth Assessments of Maize Plants

The experiment was completely randomized and organized in a 2 × 8 factorial scheme (doses × formulations) with four replicates. Each replicate was composed of a 3.5-L pot (15 cm height, 14 cm lower diameter, 18.5 cm upper diameter) containing two plants. The atrazine doses used were 1000 and 2000 g a.i. ha^–1^ for the formulations ATZ, PCL + ATZ, PCL/CS + ATZ, and ZN + ATZ. The 1000 g a.i. dose was selected based on previous studies demonstrating increased efficiency of nanoformulations in weed control. This allowed for a 50% reduction in the active ingredient applied without compromising weed control efficacy.^14^ The 2000 g a.i. dose was selected to reflect the regular field application rate. This is consistent with other field studies on weed control that utilized 2000 g a.i. of atrazine and achieved satisfactory control percentages.^24,25^

The PCL, PCL/CS, and ZN formulations were also applied at the same dilutions used for the respective herbicide formulations. As a control (CTL), maize plants sprayed only with water were used for evaluation. The formulations were applied to the leaves with a hand sprayer, using a volume of 9.4 mL per replicate.

Physiological measurements of PSII activity of the maize plants were performed at 24, 48, 72, and 96 h after application (HAA), and 7, 14, and 21 days after application (DAA). Stomatal conductance assessments were also performed at 48 and 72 HAA. At 30 DAA, morphological assessments of height, stem diameter, number of fully expanded leaves, root length, fresh mass of shoots and roots, and dry mass of shoots and roots were performed.

### Stage 2 – Physiological and Biochemical Evaluations of Maize Plants

The experiment was completely randomized, organized in a 3 × 5 factorial scheme (collection times × formulations). The collection times were 24, 48, and 72 HAA of the formulations ATZ, PCL + ATZ, PCL/CS + ATZ, and ZN + ATZ at a dose of 2000 g. a.i. ha^–1^. The CTL formulation was also adopted for plants sprayed only with water. The spray volume used was 5.1 mL pot^–1^ in a 1 L pot (10.5 cm height, 9.5 cm lower diameter, 14 cm upper diameter).

The herbicidal activity of the formulations on the plants was evaluated through physiological measurements of PSII activity and the collection of plant material for biochemical analyses of oxidative stress markers and enzymatic activities.

### Physiological Assessments

The maximum (*F*_v_/*F*_m_) and effective (ΔF/F’) quantum efficiencies of PSII were evaluated using a portable OS 1p fluorometer (Opti-Sciences, Hudson, NY, USA), which allows the detection of damage caused to PSII in plants even before visualizing phytotoxicity symptoms.^34^ The measurements were performed following the protocol described by Sousa et al.,^11^ on the fourth fully expanded leaf. The *F*_v_/*F*_m_ measurements were made between 07:30 and 09:30 h, and then, the Δ*F*/*F*′ measurements were made between 10 and 11:30 h. From the Δ*F*/*F*′ and PPFD (photosynthetic photon flux density) measurements, the relative electron transport rate (rETR) was calculated using the following equation provided by Baker:^35^

2 $$\mathrm{rETR}=\Delta F/F^{\prime }\times \mathrm{DFFA}\times 0.5\times 0.84$$

where the constants 0.5 and 0.84 correspond to the division of the energy captured by the plants between the two photosystems and the approximate value of maximum use of the light captured by the plants, respectively.

Stomatal conductance (*g*_s_) was assessed using a portable porometer SC-1 (Decagon Devices – Pullman, USA). The assessment was performed on the fourth fully expanded leaf, and consisted of attaching the equipment sensor in the form of a clip containing the chamber that measures stomatal conductance to the abaxial surface of the plant leaves, avoiding the central vein. The first assessment was conducted in the afternoon, between 2 and 4 pm, and the second was carried out in the morning, between 9:30 and 11:30 am. The measurement values were recorded after the 30 s programmed by the equipment, the time required to achieve gas stabilization inside the chamber.

### Plant Growth Assessments

At 30 days after application of the formulations, the parameters of plant height (from the base in contact with the soil), stem diameter (in the median region between the cotyledonary leaf and the sheath of the first fully expanded leaf), number of fully expanded leaves (leaves whose sheath did not touch the stem were considered and counted as fully developed), root length (considered the length of the largest root found), fresh mass of aerial parts and roots (sectioned in the transition zone present at the base of the stem, and weighed separately on a semianalytical scale), and dry mass of aerial parts and roots (after 7 days stored in paper bags in a drying oven at a temperature of 70 °C) were evaluated.

### Biochemical Evaluations

The oxidative stress in the maize plants after application of formulations containing atrazine was evaluated by quantifying substances reactive to thiobarbituric acid (TBARS method),^36^ hydrogen peroxide (H_2_O_2_),^37^ and conjugated dienes.^38^ An aliquot of 100 mg of plant material was used for each of the three analyses. The activity of enzymes with antioxidant action was evaluated from enzymatic extracts obtained by adding 1 mL of 100 mM potassium phosphate buffer (pH 7.5) containing EDTA (1 mM) and polyvinyl-polypyrrolidone (PVPP) 2% (w/v) to microtubes containing 100 mg of crushed plant material. The microtubes were shaken manually to homogenize the contents, and then centrifuged at 15,645*g*, at 4 °C for 20 min in a MIKRO 220 R refrigerated centrifuge (Hettich Zentrifugen – Tuttlingen, Germany).

For the enzyme ascorbate peroxidase (APX, EC 1.11.1.11), which uses ascorbate as a reducing agent for H_2_O_2_, the activity was determined according to the methodology of Nakano and Asada,^39^ where the consumption of ascorbate in the presence of H_2_O_2_ is monitored by absorbance at a wavelength of 290 nm. Catalase activity (CAT, EC 1.11.1.6) was determined according to Peixoto et al.,^40^ by monitoring the decrease in H_2_O_2_ through absorbance at a wavelength of 240 nm. For peroxidase activity (POD, EC 1.11.1.7) the methodology of Peixoto et al.^40^ was used, and the movement of absorbance at a wavelength of 420 nm was monitored to quantify the production of purpurogallin (result of the oxidation of pyrogallol in the presence of H_2_O_2_). The methodology of Giannopolitis and Ries^41^ was used to determine the activity of superoxide dismutase (SOD, EC 1.15.1.1), which allows for the monitoring of the extract’s efficiency in protecting nitro blue tetrazolium chloride (NBT) from photoreduction, with absorbance evaluated at a wavelength of 560 nm. The SOD activity was measured in units, where one unit of SOD corresponds to the enzymatic activity necessary to inhibit the photoreduction of NBT by 50% when compared to a control sample. Absorbance monitoring was performed using a Genesis 10S UV–vis benchtop spectrophotometer (Thermo Scientific – Madison, USA), using 4 mL quartz cuvettes for the four methodologies.

The activity of the enzyme glutathione *S*-transferase (GST, EC 2.5.1.18) was evaluated according to the protocols of Cataneo et al.^42^ The extracts were obtained from 300 mg of crushed plant material homogenized in microtubes in TRIS-HCl buffer 50 mmol L^–1^ (pH 7.0) containing 20% glycerol (v/v), ascorbic acid (1 mmol L^–1^), dithiothreitol (1 mmol L^–1^), EDTA (1 mmol L^–1^), reduced glutathione (1 mmol L^–1^), and MgCl_2_ (5 mmol L^–1^), and subsequently centrifuged at 4 °C for 6 min at 12,000*g* and at 4 °C for 16 min and 26,900*g* in a MIKRO 220 R refrigerated centrifuge (Hettich Zentrifugen – Tuttlingen, Germany). The formation of GSH–CNDB conjugates results in an increase in the absorbance of the samples, measured at a wavelength of 340 nm using a Genesis 10S UV–vis benchtop spectrophotometer (Thermo Scientific, Madison, USA) with 4 mL quartz cuvettes.

### Statistical Analysis

The *F*_v_/*F*_m_ data were transformed by arcsen√x before being subjected to statistical analysis. The normality of errors and homogeneity of variance tests were performed for all the parameters evaluated and, when these assumptions were accepted, the data were subjected to analysis of variance (ANOVA) by the F test (*p* ≤ 0.05) and subsequent mean comparison test (Tukey test *p* ≤ 0.05). All statistical analyses were performed in RStudio.^43^

## Supplementary Material



## Notes

Stomatal conductance on maize plants after treatment with atrazine formulations; plant height and root length of maize plants after treatment with atrazine formulations; size characterization, zeta potential, and atomic force microscopy (AFM) micrograph of nanocapsules containing atrazine (PDF)

## Nomenclature

a.i.: active ingredient
APX: enzyme ascorbate peroxidase
ATZ: conventional atrazine
CAT: catalase enzyme
CD: conjugated dienes
GST: glutathione *S*-transferase enzyme
H_2_O_2_: hydrogen peroxide
MDA: malondialdehyde
NCs: nanocapsules
PCL: polycaprolactone;
PCL NCs: polycaprolactone nanocapsules
PCL/CS NCs: polycaprolactone nanocapsules coated with chitosan
PCL + ATZ: polycaprolactone nanocapsules containing atrazine
PCL/CS + ATZ: polycaprolactone nanocapsule nanoformulation containing atrazine and coated with chitosan
POD: peroxidase enzyme
PSII: photosystem II
rETR: relative electron transport rate
SC: suspension concentrate
SOD: superoxide dismutase enzyme
ZN + ATZ: zein nanocapsules containing atrazine
