# Supplementary material for: Leveraging Nanotechnology for Safer Herbicide Use: Insights from Maize Tolerance to Nanoencapsulated Atrazine
Source: ACS Omega. 2025 Aug 20;10(34):38943–54. doi: 10.1021/acsomega.5c04949 (PMC12409546; doi:10.1021/acsomega.5c04949)

Supporting Information

LEVERAGING NANOTECHNOLOGY FOR SAFER HERBICIDE USE: INSIGHTS FROM MAIZE TOLERANCE TO NANOENCAPSULATED ATRAZINE

Bruno Teixeira de Sousa[1], Bruno Henrique Bortotto da Silva[2], Euler Augusto Inêz[2], Anderson do Espírito Santo Pereira[3], Jhones Luis Oliveira[3], Leonardo Fernandes Fraceto[4], Giliardi Dalazen[2], Halley Caixeta Oliveira[1]*

[1] Department of Animal and Plant Biology, State University of Londrina (UEL), 86057-970 Londrina, Paraná, Brazil.

[2] Department of Agronomy, State University of Londrina (UEL), 86057-970 Londrina, Paraná, Brazil.

[3] B.nano Technological Solutions, 18078-005, Sorocaba, São Paulo, Brazil.

[4] Institute of Science and Technology, São Paulo State University (UNESP), 18087-180 Sorocaba, São Paulo, Brazil.

B.T.S. (brunotdsousa@gmail.com), B.H.B.S. (bruno.bortotto@uel.br), E.A.I. (euler.augusto.inez@uel.br), A.E.S.P. (espdna@gmail.com), J.L.O. (jholuisoliveira@hotmail.com), L.F.F. (leonardo.fraceto@unesp.br), G.D. (giliardidalazen@gmail.com), H.C.O. (halley@uel.br)

**corresponding author*

Figure Supplementary 1. Stomatal conductance (g_s) of *Zea mays* at **(a)** 48 and **(b)** 72 hours after application (HAA) of ATZ, PCL+ATZ, PCL/CS+ATZ, or ZN+ATZ, at doses of 1000 g a.i. ha^{-1} (D1) and 2000 g a.i. ha^{-1} (D2), in addition to the nanocapsules without the a.i. in the same dilutions (PCL, PCL/CS, ZN) and control with water only (CTL). Different lowercase letters indicate differences between formulations within D1; different uppercase letters indicate differences between formulations within D2; and asterisks indicate differences between doses of the same formulation by the Tukey test $p≤0.05$.

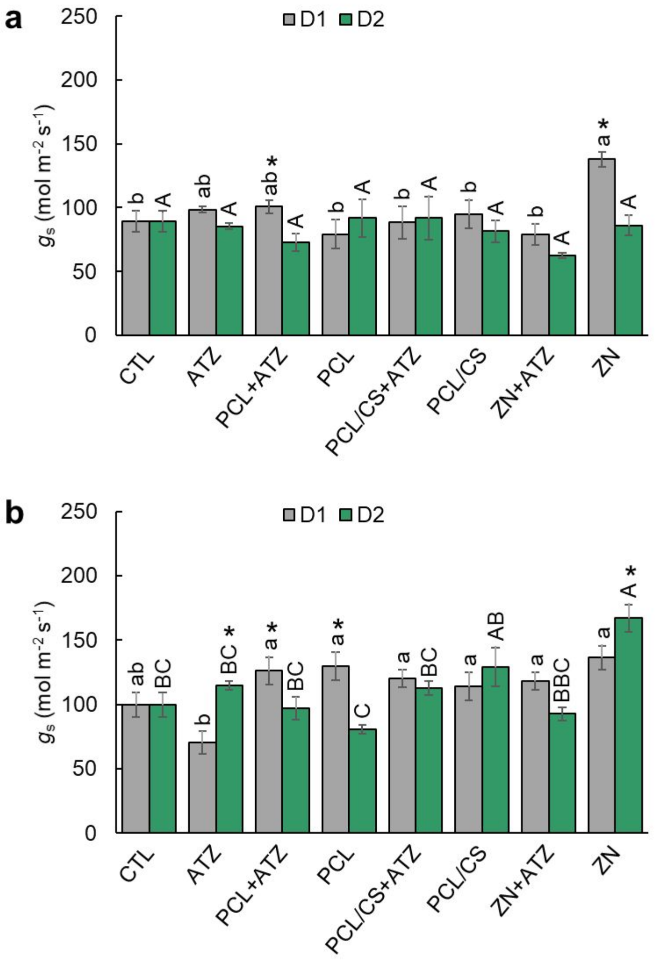

Figure Supplementary 2. (a) Plant height, and **(b)** root length of *Zea mays* 30 days after application (DAA) of ATZ, PCL+ATZ, PCL/CS+ATZ, or ZN+ATZ, at doses of 1000 g a.i. ha^{-1} (D1) and 2000 g a.i. ha^{-1} (D2), in addition to the nanocapsules without the a.i. in the same dilutions (PCL, PCL/CS, ZN) and control with water only (CTL). In **(a)**, different lowercase letters indicate differences between formulations within D1; different uppercase letters indicate differences between formulations within D2; and asterisks indicate differences between doses of the same formulation by the Tukey test $p \leq 0.05$. In **(b)**, different lowercase letters indicate differences between treatments and different uppercase letters indicate differences between doses by the Tukey test $p \leq 0.05$.

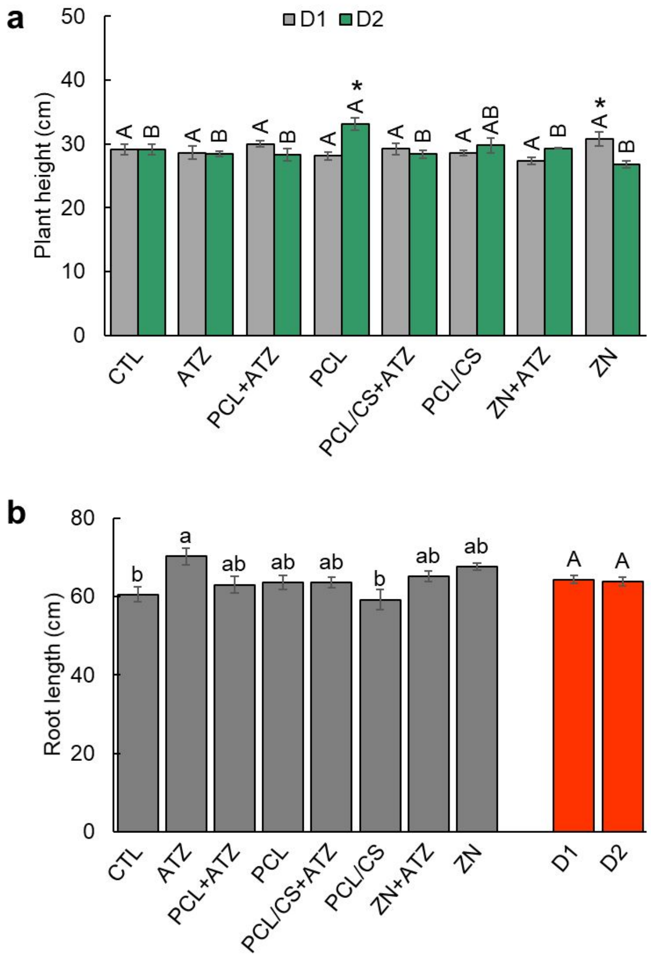

Figure Supplementary 3. Size characterization, zeta potential, and Atomic Force Microscopy (AFM) micrograph of poly(ε-caprolactone) nanocapsules containing atrazine (PCL+ATZ), PCL+ATZ nanocapsules coated with chitosan (PCL/CS+ATZ), and zein nanocapsules containing atrazine (ZN+ATZ). PCL+ATZ figures adapted from Takeshita et al.[12]; PCL/CS+ATZ figures adapted from Sousa et al.[14]; ZN+ATZ figures adapted from Carvalho et al.[13].

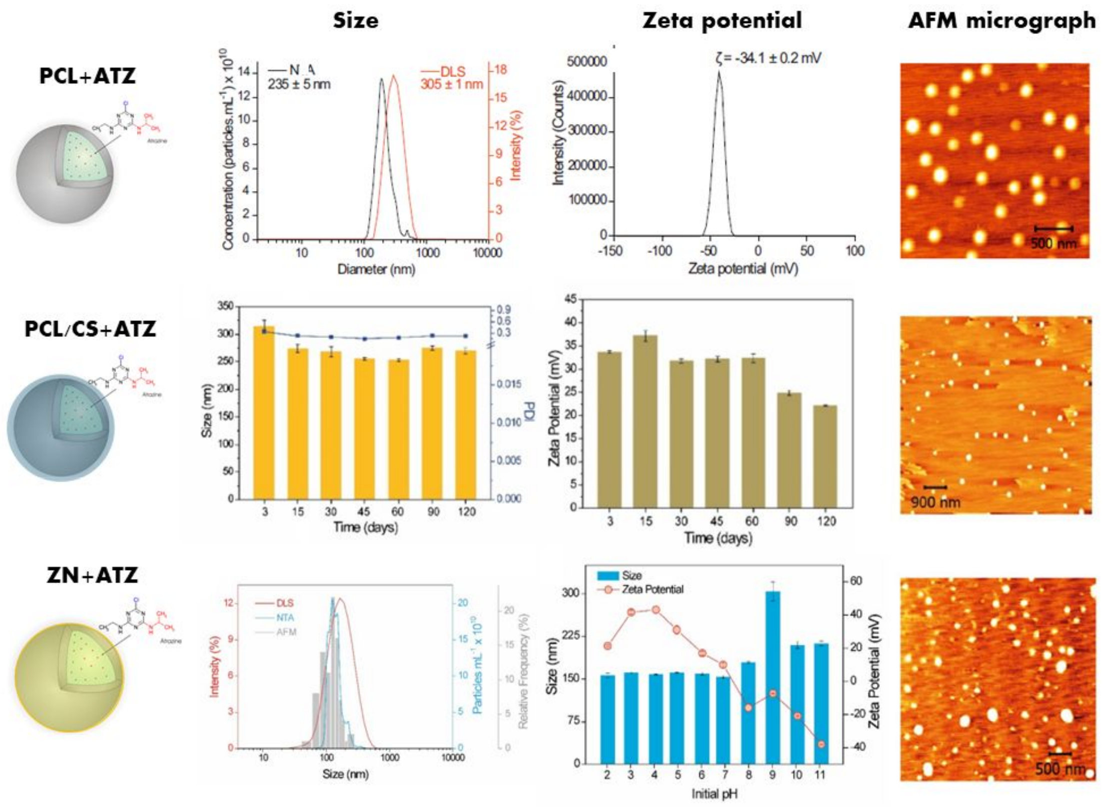

Supplement: Supplementary file 1 [file ao5c04949_si_001.pdf]
